# Development of a Static Avascular and Dynamic Vascular Human Skin Equivalent Employing Collagen/Keratin Hydrogels

**DOI:** 10.3390/ijms25094992

**Published:** 2024-05-03

**Authors:** Kameel Zuniga, Neda Ghousifam, Lucy Shaffer, Sean Brocklehurst, Mark Van Dyke, Robert Christy, Shanmugasundaram Natesan, Marissa Nichole Rylander

**Affiliations:** 1 Department of Biomedical Engineering, The University of Texas at Austin, Austin, TX 78712, USA; swbrock@gmail.com; 2 59th Medical Wing Science and Technology, JBSA-Lackland, TX 78236, USA; lucyjshaffer@gmail.com; 3 Department of Mechanical Engineering, The University of Texas at Austin, Austin, TX 78712, USA; ghosifam@gmail.com; 4 Department of Biomedical Engineering, The University of Arizona, Tucson, AZ 85712, USA; mvandyke@arizona.edu; 5 Military Health Institute, University of Texas Health San Antonio, San Antonio, TX 78229, USA; christyr1@uthscsa.edu; 6 Extremity Trauma and Amputation Center of Excellence (EACE), Defense Health Agency, San Diego, CA 92134, USA; shanmugasundaram.natesan.civ@health.mil

**Keywords:** skin, keratinocytes, differentiation, collagen, keratin, hydrogel, barrier function, vascularization, vessel, 3D culture

## Abstract

One of the primary complications in generating physiologically representative skin tissue is the inability to integrate vasculature into the system, which has been shown to promote the proliferation of basal keratinocytes and consequent keratinocyte differentiation, and is necessary for mimicking representative barrier function in the skin and physiological transport properties. We created a 3D vascularized human skin equivalent (VHSE) with a dermal and epidermal layer, and compared keratinocyte differentiation (immunomarker staining), epidermal thickness (H&E staining), and barrier function (transepithelial electrical resistance (TEER) and dextran permeability) to a static, organotypic avascular HSE (AHSE). The VHSE had a significantly thicker epidermal layer and increased resistance, both an indication of increased barrier function, compared to the AHSE. The inclusion of keratin in our collagen hydrogel extracellular matrix (ECM) increased keratinocyte differentiation and barrier function, indicated by greater resistance and decreased permeability. Surprisingly, however, endothelial cells grown in a collagen/keratin extracellular environment showed increased cell growth and decreased vascular permeability, indicating a more confluent and tighter vessel compared to those grown in a pure collagen environment. The development of a novel VHSE, which incorporated physiological vasculature and a unique collagen/keratin ECM, improved barrier function, vessel development, and skin structure compared to a static AHSE model.

## 1. Introduction

Human skin protects our body from external physical, chemical, and biological contaminants, thanks to its multiple layers and the differentiation of keratinocytes that creates a physical barrier [1,2]. As the largest organ in the body, skin covers 2 m^2^ for the average human and comprises about 15% of total adult body weight [1,2]. Therefore, many studies have been conducted to develop the most representative model for human skin to study skin diseases, wound inflammation, and the development of regenerative therapies for skin wounds [3,4,5,6,7,8,9]. 

Existing skin models consist of animal models, ex-vivo models of animal or human tissue, two-dimensional (2D) cell monolayers, or three-dimensional (3D) skin platforms lacking vasculature and immune components typically composed of only a single layer or without the addition of any 3D ECM [10,11,12,13]. Pigs are the most commonly used animal model due to the physiological similarity of their skin to human skin [4,14,15], with mice and rats being used for initial testing, but lacking in their capability to replicate the thickness, complexity, and wound healing mechanisms of human skin [14]. Although porcine skin models provide the most complete and physiologic microenvironment to human skin, the dynamics and spatial kinetics of skin injury and wound healing cannot be easily studied without significant time and expense. The majority of in-vivo assessments are also endpoint measurements (e.g., histology, RT-PCR, and immunohistochemistry (IHC)) and require an enormous number of animals to acquire information at multiple time points. In addition, in-vivo animal models do not fully recapitulate human physiology, with animal-to-animal variation that is difficult to control. Excised human skin or animal skin, often acquired during other surgeries, can be maintained alive ex vivo and experimented on [8,16,17,18]. However, its properties vary based on anatomical location and patient history or animal experimental history.

Two-dimensional cell monolayers do not recapitulate the complex 3D architecture of tissue or the inherent cell–cell and cell–matrix interactions critical for understanding response to injury and wound healing [19,20]. In addition, 2D cell monolayers are unable to express the 3D levels of differentiation of keratinocytes. Keratinocytes undergo a complex process of differentiation to develop the cornified layer. As they originate in the basal layer, keratinocytes are in a proliferative state and express cell-specific markers, such as cytokeratin 14 (CK14) [21,22,23,24]. Further up through the epidermis in the spinous layer, keratinocytes begin to express mid-differentiation markers, including cytokeratin 10 (CK10) [21], and finally become terminally differentiated and express new proteins, such as involucrin, at the beginning of terminal differentiation [25,26]. Most 3D in-vitro models consist of an extracellular matrix (ECM) seeded with fibroblasts, in which keratinocytes are located in the uppermost layer. This ECM is often in the form of collagen type I hydrogels, and currently there are no existing skin models that employ keratin [2]. Although keratin does not exist in the dermal layer, the addition of keratin in a skin model may promote the differentiation of keratinocytes. Keratin is present in keratinocytes and forms a cytoskeleton within, which resists mechanical stress. This allows the differentiation of keratinocytes into corneocytes, forming a barrier known as the stratum corneum [27]. We have previously demonstrated that keratinocytes showed increased expression of differentiation markers and lysosomal activity when seeded on a collagen/keratin blended hydrogel [28].

In-vitro skin platforms of varying complexity exist, but have been focused on creating skin substitutes for small defects rather than mimicking full thickness physiologically representative skin with vasculature for studying wound dynamics and healing. One of the primary barriers to generating full thickness physiologically representative skin is the inability to integrate fully functional vasculature with representative flow within the skin platform. The cutaneous vasculature of the skin supplies oxygen and nutrients to the dermis and the overlying epidermis, and also disposes of metabolic wastes [29]. The cutaneous vasculature is limited to the dermis, whereas the epidermis has no blood supply and receives its nutrients from the underlying dermis via diffusion [2]. When the endothelium is activated by inflammatory mediators, including vascular endothelial growth factor A (VEGF-A), tumor necrosis factor (TNF-α), and interleukin-6 (IL-6) from skin cells, there is an upregulation of adhesion molecules leading to interactions with circulating immune cells, including monocytes, which involve rolling, adhesion, and transmigration necessary for immune regulation [30,31]. In addition to providing nutrients to the skin and being involved in inflammation and wound healing processes, studies using 3D in-vitro models have shown that the presence of vasculature improves the differentiation of keratinocytes and epidermal barrier function, which is critical for replicating native skin since the dermis contains vasculature [8,11,12,13,32,33]. The presence of flow also allows the physiological transport of nutrients to the skin ECM and mechanical fluidic shear stress to promote growth and keratinocyte differentiation [12,20,34]. The flow also provides a more representative and reliable model of native skin, in terms of testing toxicity and efficacy of potential drugs [12,20,34]. With vasculature incorporated into a 3D skin culture system, one is able to incorporate an immune system with circulating immune cells and endothelial cells, allowing the investigation of the immune response and angiogenesis in the skin, respectively [33]. When compared to static cultures, 3D in-vitro models incorporating a dynamic flow demonstrated a thicker stratum corneum with an increase in epidermal differentiation marker expression, which is more physiologically representative [12,13,35]. This is likely due to dynamic perfusion inducing shear stress and mechanical stimuli that cause epidermal maturation and may control its barrier function [13,36,37]. In addition, a dynamic flow creates a pressure gradient throughout tissue, whereas transport of nutrients only occurs through diffusion in static culture [13]. Since dynamic flow occurs in vitro with the presence of blood vessels in the skin, it is important to mimic this in in-vitro models to create a more realistic model to allow future transport studies in response to certain drugs (chemical) and thermal and biological stimuli with the incorporation of the immune response with circulating immune cells. However, current models have not incorporated a functional blood vessel with endothelial cells, which may affect overall perfusion of nutrients and overall keratinocyte differentiation and cornification. 

In this current study, we created a unique vascularized HSE (VHSE) model employing collagen/keratin (C/KTN) hydrogels to better mimic native ECM and the incorporation of functional vasculature with a physiological flow. Our previous study creating C/KTN hydrogels with keratinocytes seeded as a 2D monolayer demonstrated that KTN increased the expression of certain differentiation markers, including involucrin [28]. We also employed a collagen hydrogel that mimicked the stiffness of healthy tissue, which has been characterized previously for its mechanical properties and promoted the growth of normal human dermal fibroblasts [38]. To create a physiologically representative vascularized HSE, we must determine whether keratin and a dynamic culture with vasculature induce differentiation and formation of a multi-layered epidermal structure that is more representative of native skin to avascular static models using transwells in terms of barrier function, keratinocyte differentiation, and overall architecture. A more representative skin equivalent would be beneficial to the study of skin diseases, wound healing, melanoma metastasis, and the immune response [39]. This was achieved by creating a static organotypic skin model employing a transwell system and advancing the complexity to the VHSE. We first created avascular static human skin equivalents (AHSEs) using transwells with a fibroblast feeder layer to serve as the dermis and keratinocytes seeded on top. This was done to determine whether KTN optimized the barrier function of the stratified epidermal layer by measuring the dextran perfusion and transepithelial electrical resistance. Finally, we created the VHSE with a functional endothelialized vessel. The vessel of the VHSE was perfused with a physiological flow to determine whether a dynamic culture promoted keratinocyte differentiation by achieving a thicker epidermis and improved barrier function, with results compared to the avascular model. 

## 2. Results

### 2.1. Barrier Function of AHSE and VHSEs

**Figure 1:**
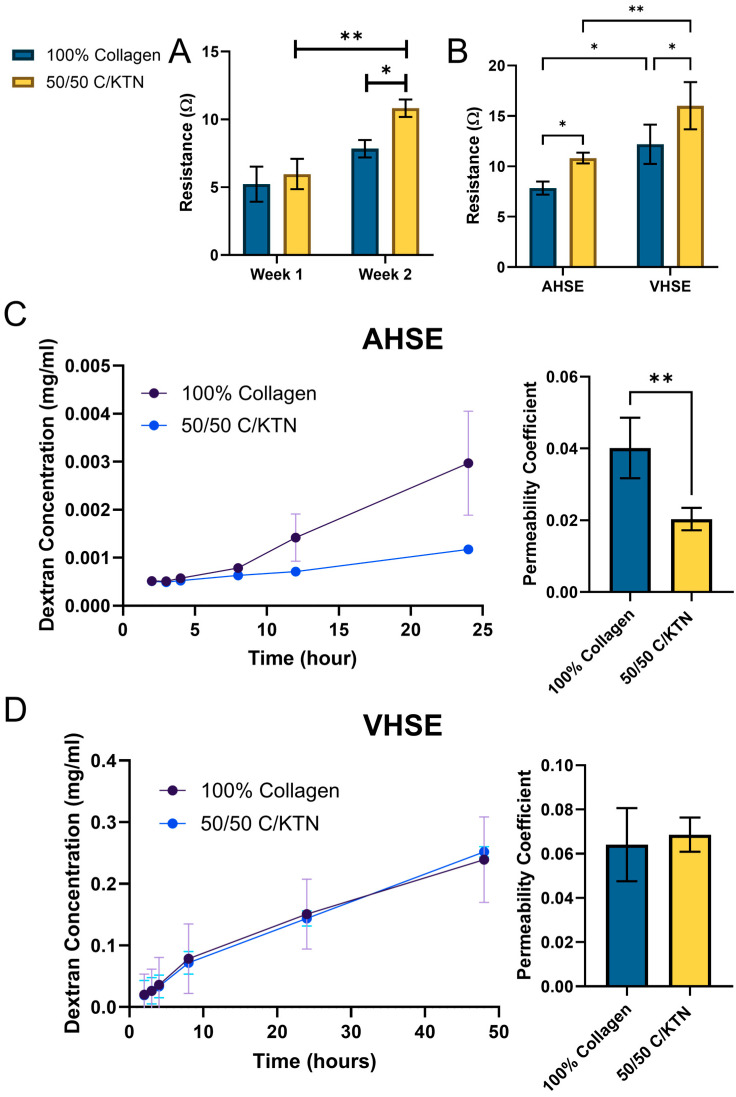
Barrier function of AHSE and VHSE. (**A**) Transepithelial electrical resistance (TEER) of avascular models at week 1 and 2 was measured, with significantly higher resistance measured at week 2. (**B**) 100% collagen and 50/50 C/KTN samples of avascular and vascular models were measured with TEER and had significantly higher resistance observed with 50/50 C/KTN avascular and vascular models, and significantly higher resistance with vascularized HSEs than AHSEs. Dextran perfusing through the barrier of (**C**) avascular and (**D**) vascular HSEs was measured over time and plotted in the linear region (left) to calculate the permeability coefficient (right). A significantly lower permeability coefficient was measured with 50/50 C/KTN compared to 100% collagen AHSEs. No significant differences were observed for vascular HSEs. A two-way ANOVA and *t*-test were performed with * = *p* < 0.05 and ** *p* < 0.01; *n* = 3 or 4.

Comparisons between week 1 and week 2 of AHSEs showed a significant increase (*p* < 0.01) in resistance for 50/50 C/KTN hydrogels, whereas 100% collagen hydrogels showed no significant differences for the time points (Figure 1A). TEER measurements also showed a significant increase in resistance (*p* < 0.05) measured for 50/50 C/KTN hydrogel models (10.827 ± 0.528 Ω for AHSE; 16.013 ± 2.341 Ω for VHSE) compared to collagen hydrogels (7.84 ± 0.647 Ω for AHSE; 12.191 ± 1.950 Ω for VSHE), with significant increases (*p* < 0.05 for 100% collagen; *p* < 0.01 for 50/50 C/KTN) with VHSEs when compared to AHSEs (Figure 1B). The permeability coefficient was also determined by measuring the fluorescence intensity of the solution underneath the transwell and the vascularized platform. As shown in Figure 1C,D on the left the dextran concentration was plotted over time and the slope of the linear region was used to calculate the permeability coefficient (Figure 1C,D, right). The permeability coefficient of the AHSE composed of 100% collagen hydrogel (0.04 ± 0.008) was significantly higher (*p* < 0.01) than that of 50/50 C/KTN hydrogels (0.02 ± 0.003), indicating a stronger barrier created with the 50/50 C/KTN hydrogel avascular model. There were no significant differences between 100% collagen (0.064 ± 0.017) and 50/50 C/KTN (0.069 ± 0.008) VHSEs.

### 2.2. Vessel Permeability of VHSE

**Figure 2:**
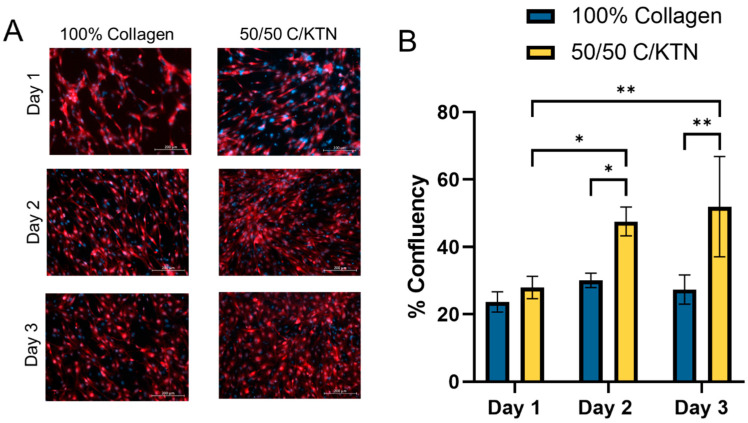
TIME cells seeded on hydrogels. (**A**) Samples were imaged at days 1, 2, and 3 post-seeding to observe cell structure and measure confluency. (**B**) Cells appear to become smaller with higher confluency over time. % confluency was measured using ImageJ software (Windows ij45-win-java8), with significantly higher confluency observed at days 2 and 3 post-seeding with 50/50 C/KTN hydrogels. A two-way ANOVA was performed with * = *p* < 0.05 and ** *p* < 0.01; *n* = 3 or 4. Scale bar = 200 µm.

To determine whether there was an increase in proliferation and growth of endothelial cells on 50/50 C/KTN hydrogels compared to pure 100% collagen gels, telomerase-immortalized microvascular endothelial (TIME) cells were seeded on hydrogels as a monolayer and imaged over the course of 3 days. TIME cells have previously been used in our lab to develop 3D vascularized in-vitro tumor models, in which we were able to demonstrate, in the absence of tumor cells, that TIME cells were able to form a tight vessel without any gaps, which was confirmed through CD31 staining and SEM imaging [40,41,42,43]. As shown in Figure 2A, after day 1 post-seeding, TIME cells exhibited greater confluency over time on 50/50 C/KTN hydrogels compared to 100% collagen hydrogels The confluency was calculated through ImageJ software (Windows ij45-win-java8)and it was confirmed that cell confluency significantly increased over time (*p* < 0.01) when TIME cells were seeded on top of 50/50 C/KTN hydrogels, whereas there was no significant difference over time with TIME cells seeded on top of 100% collagen hydrogels (Figure 2B). In addition, the confluency was significantly higher with cells seeded on top of 50/50 C/KTN hydrogels (47.57 ± 4.25% (day 2); 51.98 ± 14.88% (day 3)) when compared to 100% collagen hydrogels (30.11 ± 2.11% (day 2); 27.384 ± 4.328% (day 3)) at days 2 (*p* < 0.05) and 3 (*p* < 0.01). 

**Figure 3:**
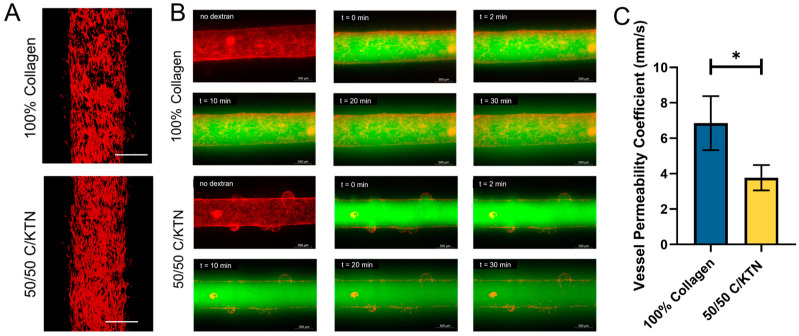
Skin vessel structure and permeability of VHSE. (**A**) The vessel formed in the VHSE was imaged with confocal microscopy and exhibited larger gaps in the 100% collagen hydrogel vessel. (**B**) 0.1 mg/mL 70 kDa dextran was injected through the vessel of the VHSE and images were taken every 2 min to measure the fluorescence intensity over time. (**C**) The vessel permeability coefficient was calculated with fluorescence intensity measurements outside the vessel over a course of 30 min, with a significantly lower permeability coefficient observed with the 50/50 C/KTN VHSE. A t-test was performed with * = *p* < 0.05, *n* = 3. Scale bar = 500 µm.

TIME cells were able to form a vessel with a dynamic flow in both 100% collagen and 50/50 C/KTN vascular HSEs, as shown in Figure 3A. To determine whether there were any differences in permeability between the vessel formed inside the 100% collagen hydrogel and the 50/50 C/KTN, the permeability coefficient was determined by measuring the fluorescence intensity of the dextran that diffused outside the vessel in a ROI in images taken every 2 min over the course of 30 min (Figure 3B). A significant decrease (*p* < 0.05) in the permeability coefficient of the vessel was observed when formed in 50/50 C/KTN hydrogels (3.77 ± 0.72) when compared to the vessel in 100% collagen hydrogels (6.86 ± 1.52), confirming that TIME cells form a more confluent and less leaky vessel on top of and inside a 50/50 C/KTN hydrogel (Figure 3C). 

### 2.3. Skin Structure of AHSE and VHSEs

**Figure 4:**
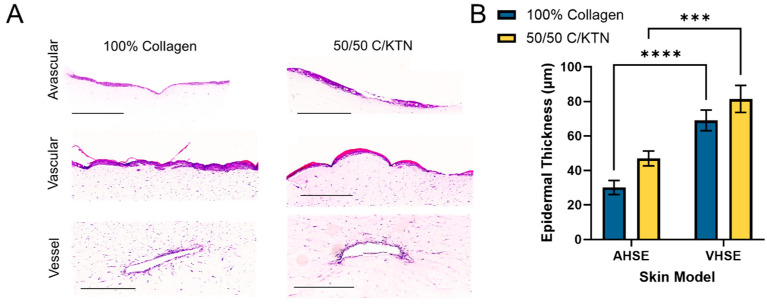
H&E staining of avascular and vascular models. (**A**) Samples were processed, sectioned, and stained with H&E and imaged to observe the epidermal structure and the vessel. (**B**) Images were used to measure epidermal thickness, with thickness increasing significantly with the vascular HSE. A two-way ANOVA was performed with *** *p* < 0.001 and **** *p* < 0.0001; *n* = 15. Scale bar = 500 µm.

H&E staining confirmed the formation of a multilayered epidermal structure, with a well-defined stratum corneum layer (pink) observed with the vascularized model, as shown by the representative images in Figure 4A. Both the 100% collagen and 50/50 C/KTN VHSE also successfully showed the presence of cells outlining the vessel wall. The measured epidermal thickness was significantly greater (*p* < 0.0001) for the VHSE (69.07 ± 23.23 μm (100% collagen); 81.44 ± 30.31 μm (50/50 C/KTN)) compared to the AHSE (30.12 ± 15.96 μm (100% collagen); 46.96 ± 16.90 μm (50/50 C/KTN)) as shown in Figure 4B, indicating that the presence of dynamic flow and vasculature can create a thicker epidermal structure. Although no significant differences in epidermal thickness were observed between the 100% collagen and 50/50 C/KTN hydrogels, there was a trend in the increase of the thickness with the addition of KTN in the hydrogel (Figure 4B). The thickness of the human epidermis can range anywhere from 50 to 250 μm depending on the location of the body [44]. Therefore, the VHSE is more representative of human skin in regard to epidermal thickness.

### 2.4. Differentiation Marker Expression in AHSE and VSHEs

**Figure 5:**
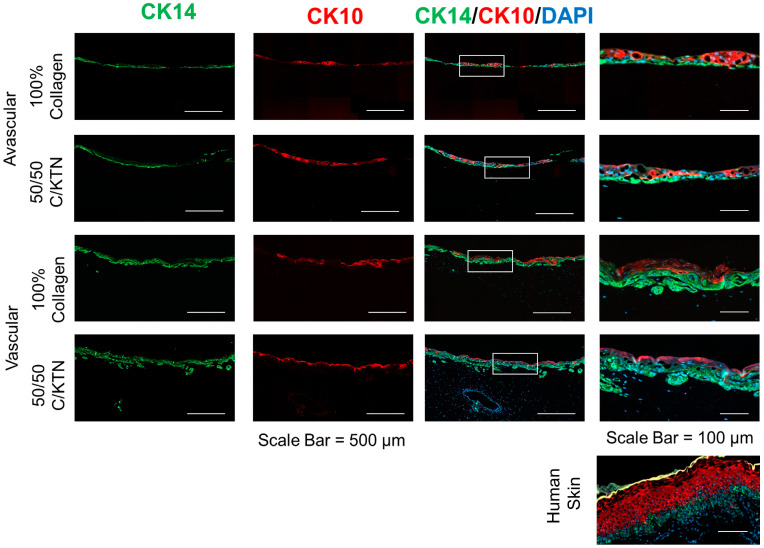
IHC staining of CK14 and CK10 for avascular and vascular HSEs. CK14 and CK10 were expressed in both avascular and vascular HSEs, with a thicker layer of CK14 expression throughout the whole epidermal layer underlying CK10-expressing keratinocytes with the vascularized model, similar to what was observed in native human skin. Farthest right images are magnified areas of the images on the left in the white highlighted box.

**Figure 6:**
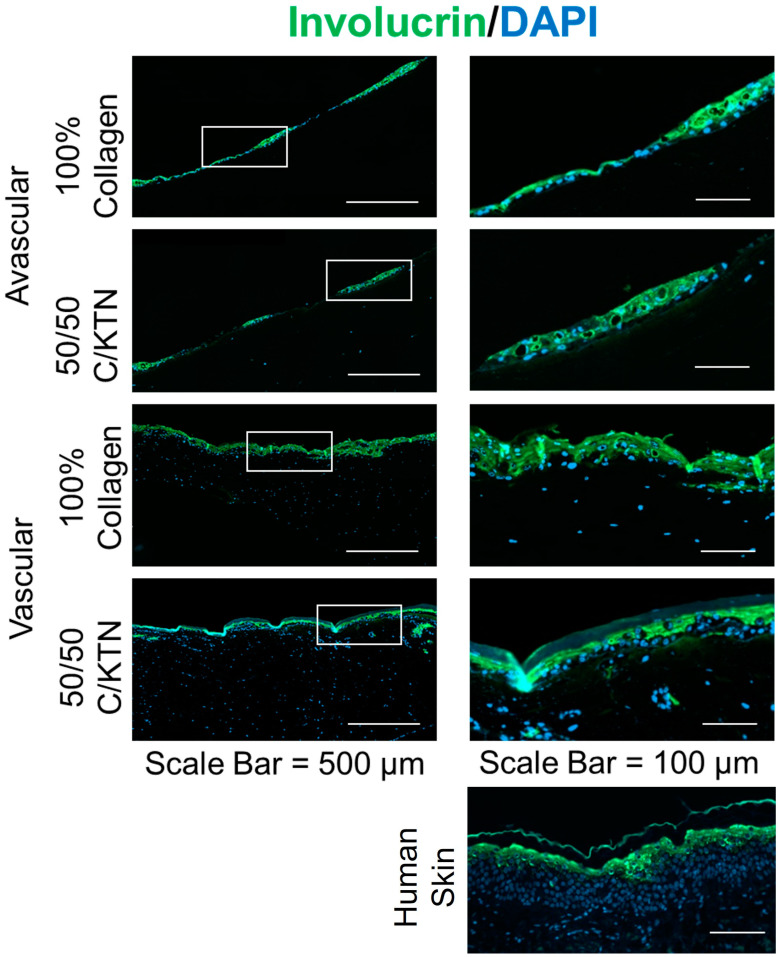
IHC staining of involucrin for avascular and vascular HSEs. Involucrin was expressed in both AHSEs and VHSEs, although there was expression throughout the whole epidermal layer with the vascularized model, similar to what was observed in native human skin. Farthest right images are magnified areas of the images on the left in the white highlighted box.

Avascular and vascular HSEs were fixed and stained for differentiation markers, including CK14, CK10, and involucrin, to determine if keratinocytes showed differences in the differentiation between avascular and vascular HSEs. As shown in Figure 5 both CK14 and CK10 were expressed in all samples, which signifies the differentiation of keratinocytes, regardless of the hydrogel formulation and the presence of a vessel. Similarly, as shown in Figure 6 involucrin, which signifies terminal differentiation, was also expressed in all samples. However, the layer of CK14 expression (basal layer) in the AHSE was much thinner than VHSE, with CK10 not expressed throughout the whole layer of the samples. This indicates that the basal layer (CK14) in the AHSE was not proliferating as much as the VHSE, with minimal and patchy differentiation (CK10) of keratinocytes compared to the VHSE. Similarly, with CK10, involucrin was not expressed throughout the whole layer of the AHSE as shown in Figure 6, whereas a continuous layer of involucrin expression was observed with the VHSE, meaning keratinocytes were differentiating throughout the whole layer of the epidermis. Therefore, the vascularized model allows greater proliferation of basal keratinocytes and the development of the basement membrane with higher and homogenous differentiation of keratinocytes, which develop into the stratum corneum layer.

## 3. Discussion

In this present study, we successfully created a VHSE that possessed increased keratinocyte differentiation and barrier function compared to the AHSE that was exposed to static conditions. This was observed by the expression of basal and differentiation markers (CK14, CK10, and involucrin) throughout the whole layer of the epidermis in our VHSE, whereas these markers were expressed irregularly in the AHSE model. In our AHSE, we were also able to show that the inclusion of keratin improved the barrier function significantly compared to collagen-only ECM, comparable to our previous study [28]. Through the VHSE model, we were also able to perfuse dextran through the vessel, mimicking nutrient diffusion and overall permeability of the vessel over time. Keratinocytes are the optimal cells to use for skin models and are closest physiologically to native skin; however, they are difficult to culture long-term with limited availability of fresh donor skin and donor-to-donor variation [45,46]. The use of telomerase-immortalized keratinocytes (Ker-CT) in our study can overcome these obstacles of creating a 3D in-vitro vascularized model with high throughput capability without donor-to-donor variation. Therefore, we were able to avoid the sample-to-sample variation when creating these HSEs. In addition, the use of a small platform with minimal materials, including collagen and keratin hydrogel, and differentiation allows high throughput and a reduction in supply costs over time. 

Most organotypic skin models use collagen as the ECM for the dermal layer, in which fibroblasts are seeded in and keratinocytes are seeded on to form the epidermis [19,47,48]. Keratin is the main protein present in the epidermis since it is produced by keratinocytes, making up almost 90% of the epidermis [2]. Although keratin is not present in the dermal layer, we wanted to test whether keratin promotes the differentiation of the keratinocytes in which they are seeded. Because keratin is naturally found in the epidermis, we employed a 50/50 w%/w% collagen/keratin hydrogel, which allowed the spreading and proliferation of NHDFs as previously shown by the Rylander lab [38].

Avascular models showed that the addition of KTN in the hydrogel also improved barrier function. The fluorescence intensity of dextran that diffused through the epidermis was significantly lower with 50/50 C/KTN avascular models than with 100% collagen hydrogels with a decreased permeability coefficient, meaning fewer dextran particles were able to permeate through the barrier. Similarly, resistance measured through TEER increased significantly compared to 100% collagen hydrogels, indicating that KTN may have possibly increased the differentiation of keratinocytes. This may be explained by the presence of cell-binding motifs, including LDV and EDS, on extracted keratin. LDV has been show to promote adhesion of skin-related cells, including keratinocytes, which could possibly lead to the establishment of a better defined basement membrane [49]. Cell–cell adhesion is critical for the formation of an intact and multilayered epidermis by the formation of adherens junctions, desmosomes, and tight junctions [50]. Without an initial attachment to the ECM, these cell–cell adhesion points are difficult to form, leading to poor barrier function and mechanical robustness [50]. However, more importantly, the inclusion of vasculature in the model showed that there was increased resistance measured through TEER compared to the static AHSE model, affirming that the vasculature and flow increased the barrier function of the epidermis. Similar results were also observed with TEER, in which flowing media increased the resistance of the epidermis compared to static media in an organotypic skin model [13].

The formation of a vascularized HSE proved to be challenging, as a hollow vessel within a hydrogel ECM needed to remain stable for 2 weeks of differentiation. In addition to having an open system, in which collapse of the hydrogel was more likely to happen than one in a closed system, the vessel could have collapsed as well. Therefore, placing the vascularized HSE on a rocker in the incubator proved to keep the vascularized HSE more stable than if we had provided a continuous flow with a syringe pump, vulnerable to pressure gradients and collapse. Within a rocker system, the media was able to perfuse in and out of the vessel, mimicking blood flow. In addition, the seeding of TIME cells inside a hollow channel surrounded by 50/50 C/KTN showed increased confluency and significantly decreased permeability of 70 kDa dextran than 100% collagen. There is a possibility that this may be due to the differing porosity between 100% collagen and 50/50 C/KTN hydrogels. However, previous studies by our lab have confirmed that there are no significant differences in porosity between low-concentration (3 mg/mL) 100% collagen and 50/50 C/KTN hydrogels [38]. Even in the 2D monolayer model, TIME cells attached and proliferated more when seeded on top of 50/50 C/KTN hydrogels with higher confluency than TIME cells seeded on top of 100% collagen hydrogels. As discussed earlier, this may be due to the increased cell-binding motifs present from the KTN.

The inclusion of vasculature or flow has been shown to improve barrier function and increase epidermal thickness in previous studies [5,6,20]. Through H&E staining, a multilayered and thicker epidermis can be clearly observed with a dynamic flow, with the measured epidermal thickness significantly greater than that of samples in the static culture. In a previous study by Sriram et al., the inclusion of a flow into their transwell organotypic skin model created a thicker epidermal layer compared to their static control, similar to what we observed in our study [13]. This may be due to the increased interstitial flow due to a continuous flow of media through the vessel compared to a static system [13]. The dynamic perfusion may also induce shear stress, providing mechanical stimulation and maturation of epidermal keratinocytes [36]. In addition, it was difficult to observe a continuous stratum corneum layer with a static culture, compared to a dynamic culture with a functional vessel. No significant differences were observed in epidermal thickness between 100% collagen and 50/50 C/KTN samples, even though there was an increasing trend in epidermal thickness with the addition of KTN. This may be due to the dynamic culture of the vessel masking the effect of the KTN influence on keratinocyte differentiation. In addition, 2 weeks of culture at the ALI will most likely change the overall ECM structure, with fibroblasts continuously remodeling the ECM. It is reported that the thickness of the human epidermis can range anywhere from 50 to 250 μm, depending on the location of the body [51]. This is closer to what was observed in our VHSE model, whereas the AHSE model had an epidermis that was thinner than 50 μm. Therefore, the VSHE creates a better representation of the native human epidermis. In addition, a dynamic culture with a constant flow has been shown to increase the viability of keratinocytes in culture, allowing the longer cultivation of the 3D skin system and long-term studies [32,35].

We were able to demonstrate that all three markers of differentiation (CK14, CK10, and involucrin) were clearly expressed and distinct in the vascularized HSE, whereas in the avascular model, individual layers were difficult to distinguish from the IHC staining. The layer of CK14-positive cells, representative of proliferating cells in the basal layer, was thicker and well organized in the vascularized HSE, whereas in the AHSE, the layer was thin (1–2 cell layers) and not continuous, indicating that there was no proper development of the basement membrane of the epidermis. This is comparable to native human skin, in which a continuous layer of multiple cell thickness was observed expressing CK14 [52]. In addition, proper differentiation did not occur in the AHSEs because continuous expression of CK10 and involucrin was not observed, whereas vascularized HSEs showed expression of these differentiation markers throughout the entire section of the top epidermal layer. Similar findings were observed in previous studies involving flow-mediated skin models, in which higher expression and well-differentiated layers of differentiation markers were observed compared to a static model [13]. Sriram et al. expressed similar findings with their skin model with induced flow in dynamic conditions, in which a higher expression of involucrin and CK10 was observed compared to a static model [13]. However, there were no observable changes between 100% collagen and 50/50 C/KTN avascular or vascular HSEs. There may not be observable differences until the level of differentiation is quantified. In the future, the proper quantification of these differentiation markers is needed with further investigations to determine the proper flow settings to mimic blood flow. These further studies will enable the creation of a VHSE with better differentiation comparable to human skin. 

## 4. Materials and Methods

### 4.1. Collagen Extraction

Collagen type I was isolated from rat tail tendons in a 1N HCl solution (Fisher Scientific, Hampton, NH, USA) at an approximate pH of 2.0 and was stirred for 16 h at room temperature, as published previously [28,38,40,41,42,43]. The solution was then transferred to 50 mL centrifuge tubes (Fisher Scientific, Hampton, NH, USA) and centrifuged at 30,000× *g* (16,000 rpm) for 60 min at 4 °C to pellet the insoluble components. The supernatant was decanted from each tube and transferred into separate 50 mL centrifuge tubes, stored at −20 °C overnight, and freeze-dried for 48 h. Samples were dissolved in an appropriate amount of 0.01% glacial acetic acid (Fisher Scientific, Hampton, NH, USA) for a final concentration of 8 mg/mL of collagen stock solution. 

### 4.2. Keratin Extraction

KTN was kindly donated by Dr. Mark Van Dyke and was extracted from human hair obtained from a commercial source. Briefly, human hair was washed, chopped into small pieces, and soaked in a solution of 0.5 M thioglycolic acid (Sigma-Aldrich, St. Louis, MO, USA) in deionized (DI) water for 12 h at 36 °C, with gentle stirring [53]. Hair was then filtered from the liquid with a 500 µm sieve (W. S. Tyler, Mentor, OH, USA) and the reducing solution was retained. Free proteins were further extracted in an excess of 100 mM Tris base for 1 h, followed by DI water for 1 h with gentle shaking at 37 °C. Extracts were collected with a 500 µm sieve and combined with the reductant solution. This entire process was repeated an additional time, and all extracts were combined, centrifuged, and filtered. The combined extracts were then purified and concentrated using tangential flow filtration, were frozen, and were lyophilized. 

### 4.3. Cell Culture

All cell types were seeded in T-75 Eppendorf HEPA-filtered flasks (Eppendorf, Hamburg, Germany). Normal human dermal fibroblasts (NHDFs, passage 4–9) were cultured in a complete fibroblast media composed of fibroblast basal medium 2 (PromoCell, Heidelberg, Germany), supplemented with 2% fetal calf serum, 0.1% human fibroblast growth factor, 0.5% human insulin (PromoCell, Heidelberg, Germany), and 1% penicillin–streptomycin. Telomerase-immortalized epidermal keratinocytes (Ker-CT cells, passage 30–35, ATCC, Manassas, VA, USA) were cultured in a complete proliferation media composed of keratinocyte growth medium 2 (PromoCell, Heidelberg, Germany), supplemented with 0.004 mg/mL bovine pituitary extract (BPE), 0.125 ng/mL recombinant human epidermal growth factor (EGF), 5 µg/mL recombinant human insulin, 0.33 µg/mL hydrocortisone, 0.39 µg/mL epinephrine, 10 µg/mL recombinant human transferrin, and 0.06 mM CaCl_2_. This media formulation will be denoted as the proliferation media. When cultured for differentiation, Ker-CT cells were cultured in the proliferation media formulation without bovine pituitary extract (BPE) and epidermal growth factor (EGF), with 10% fetal bovine serum (FBS), 2.5 mM CaCl_2_, and 0.05 µg/mL ascorbic acid. BPE is documented to promote keratinocyte growth and proliferation. The calcium concentration was increased in this media (differentiation media) because an increase in calcium concentration is documented to increase differentiation in native skin, as well as in numerous skin model studies [22,54,55,56,57,58]. TIME cells were cultured in a complete endothelial media composed of 2% FBS, 0.04% hydrocortisone, 0.4% human basic fibroblast growth factor, 0.1% vascular endothelial growth factor, 0.1% R3-insulin-like growth factor-1, 0.1% ascorbic acid, 0.1% human epidermal growth factor, 0.1% gentamicin, amphotericin B, and 0.1% heparin (Lonza, Basel, Switzerland). All cells were maintained in 5% CO_2_ atmosphere at 37 °C in a sterile cell-culture incubator and corresponding complete media was changed every 2 days. Cells were detached from the flask by washing with 1× phosphate buffered saline (PBS) and replaced with 4 mL of accutase (PromoCell, Heidelberg, Germany) for NHDFs and Ker-CT cells, and 3 mL 0.25% trypsin and 0.1% EDTA in Hank’s balanced salt solution (HBSS) (Mediatech, Inc., Manassas, VA, USA) for TIME cells, followed by incubation for 3 min for NHDFs and TIME cells and 15 min for Ker-CT cells. The trypsin/cell solution was neutralized with corresponding complete media, transferred to a 15 mL conical tube, and centrifuged at 200 g for 3 min for 5 min. The pellet was isolated and resuspended in 1 mL of complete media for cell-counting. 

### 4.4. Collagen/Keratin Hydrogel Fabrication

The properties of healthy skin tissue were simulated using 4 mg/mL collagen solution, as done in previous studies [59,60,61,62]. A total of 4 mg/mL collagen was chosen to mimic healthy tissue, in which we have characterized the material and cell growth properties in a previous study [28,38]. One hundred percent collagen hydrogels were prepared from 8 mg/mL collagen stock solution and mixed, and neutralized with 10× DMEM (Sigma-Aldrich, St. Louis, MO, USA), 1× DMEM (Gibco™, Gaithersburg, MD, USA) and 1 N NaOH (Fisher Scientific, Hampton, NH, USA) for a resulting pH of 7.4, following a recipe used previously which is based on the collagen concentration of the stock solution. Fifty/fifty w%/w% C/KTN hydrogels were prepared by combining stock collagen and KTN dissolved in a neutralizing buffer of equal volume, following a similar protocol established in previous studies [28,38]. For example, to create a 50/50 C/KTN hydrogel, 8 mg/mL was mixed with 8 mg/mL collagen stock solution, which created a 4mg/mL solution of collagen and keratin each. Stock collagen and KTN/neutralizing buffer solution were combined at 1:1 *v*:*v* ratio and mixed thoroughly with a spatula and a positive displacement pipette on ice. Because the NHDFs were embedded and seeded within the hydrogel, the hydrogel-cell solution was made by centrifuging and isolating the appropriate number of cells, adding the non-polymerized hydrogel mixture directly to the cell pellet, and mixing thoroughly with a positive displacement pipette. The solutions were added and allowed to polymerize in the appropriate platform, as described in the following sections.

### 4.5. AHSE Model Fabrication 

**Figure 7:**
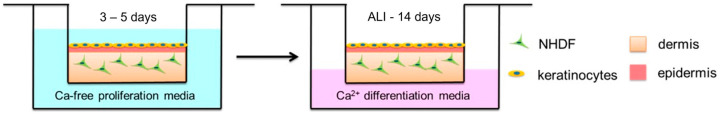
Schematic of avascular organotypic skin model fabrication utilizing a transwell. After culturing keratinocytes on top of the collagen or C/KTN layer until reaching 90% confluency, a calcium-free media was replaced with a Ca^2+^-positive differentiation media and lifted to the ALI for 14 days.

Transwell avascular HSEs (AHSEs) were made to determine the effect of KTN with the combination of the fibroblast feeder layer and the air-liquid interface on the level of keratinocyte differentiation, as well as barrier function of the developed stratum corneum. First, 600 µL of either 100% collagen or 50/50 C/KTN hydrogel solution seeded with 0.1 × 10^6^ NHDFs/mL were incorporated onto 12 mm transwell inserts with a 3.0 µm polycarbonate membrane (Fisher Scientific, Hampton, NH, USA) as the dermal layer. AHSEs were immersed and cultured in a fibroblast media overnight. On the next day, the fibroblast media was replaced with a proliferation media and cultured for one hour to rinse the calcium out of the hydrogels. Subsequently, the media was aspirated and 0.05 × 10^6^ keratinocytes in proliferation media were seeded on top of hydrogels. The proliferation media was also placed underneath the transwell in the well itself to immerse the hydrogel. After culturing the AHSEs for 3–5 days, until keratinocytes reached 90% confluency, the proliferation media was replaced with 500 µL of differentiation media underneath the AHSE and the top of the hydrogel was lifted to the air-liquid interface (ALI), as shown in Figure 7. AHSE models were cultured for 2 weeks, and the media was changed every 2–3 days before analysis. 

### 4.6. VHSE Model Design and Fabrication

**Figure 8:**
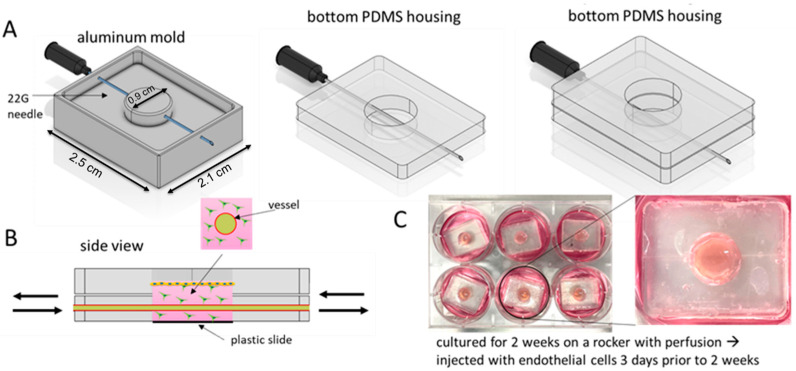
Schematic of fabrication of the VHSE utilizing a PDMS mold. (**A**) An aluminum mold is used to create the PDMS housings, which are then bonded together by plasma treatment. (**B**) A side view of the vascular HSE composed of the NHDF-seeded dermal hydrogel layer, with the hollow vessel running through and keratinocytes seeded on top. (**C**) Vascular HSEs are cultured in 6-well plates at the ALI and placed on a rocker for 2 weeks to allow media exchange through the vessel.

VHSEs were fabricated using Polydimethylsiloxane (PDMS) housing components interfaced with a plastic coverslip (Ibidi, Fitchburg, WA, USA), bonded through plasma treatment. The aluminum molds used to fabricate the PDMS housing components were computer numerical control or CNC-machined, and provided an opening on each side to hold a 22G needle, as shown in Figure 8. To create the PDMS housing components, 10:1 *w*/*w* ratio of PDMS to PDMS curing agent (SYLGARD elastomer kit, DOW, Midland, MI, USA) was poured into the aluminum mold, with a needle inserted into the mold. The molds were cured for a minimum of 1 h in the oven at a 60 °C. The PDMS housing components were removed from the molds; the bottom mold was bonded with a plastic coverslip, and the top mold was bonded to the bottom mold membrane by plasma treatment and placed in the oven overnight to allow stronger bonding.

PDMS housing components were first treated with 0.1% polyethyleneimine (PEI) (Sigma-Aldrich, St. Louis, MO, USA) and 0.5% glutaraldehyde (Sigma-Aldrich, St. Louis, MO, USA), following a similar protocol established in the Rylander lab [40,43], and were subsequently rinsed with water. The PDMS housing was treated so the collagen more effectively adhered to the PDMS surface. The bottom component of the platform was injected with 400 μL of 4 mg/mL collagen or 50/50 C/KTN hydrogel solution seeded with 0.1 × 10^6^ NHDFs/mL to serve as the dermal layer (Figure 8B). An additional 100 μL of the hydrogel solution was pipetted into the top compartment. A 22-gage needle was then inserted into the inlet of the bottom layer to create a hollow 700 µm hollow to mimic a blood vessel. The platform was polymerized for 1 h at 37 °C in a sterile incubator supplied with 5% CO_2_. Once polymerized, the needle was removed, and the platform was incubated with NHDF complete media in a 6-well plate overnight. 

After incubating overnight, the VHSEs were incubated for 1 h with fresh proliferation media to dilute the calcium present in the NHDF media. This was done to prevent the inhibition of keratinocyte proliferation. Subsequently, Ker-CT cells were seeded on top of the platform in the upper compartment in the proliferation media at a seeding density of 0.4 × 10^6^ cells/sample. The keratinocytes were cultured for 2–3 days until reaching 100% confluency and the media was removed from the top compartment to differentiate Ker-CT cells at the air–liquid interface. The differentiation media was injected into the vessel and 1.5–2 mL of differentiation media was placed in each well of the 6-well plate surrounding the platform. Plates containing platforms were placed on a rocker to allow the media to flow in and out of the vessel to mimic blood flow (Figure 8C). Platforms were cultured for 2 weeks at 37 °C in a sterile incubator supplied with 5% CO_2_. The media was changed every 2–3 days. 

Three days prior to the 2-week time point of incubation, 20 µL of 10 × 10^6^ TIME cells/mL in complete endothelial cell media were injected into the channel of the platforms, following a protocol established by the Rylander lab [40,41,42,43]. Once injected, the platforms were rotated on all 4 sides for 4 min per rotation so the cells could attach on the entire surface of the hollow vascular channel. A second injection of cells was inserted, and the process was repeated. All platforms were then cultured at the ALI in 6 well plates, with 1.5–2 mL of complete endothelial cell media placed in each well surrounding the platform. The plates were placed back on the rocker and cultured for 3 days to allow formation of a confluent vessel. The media was changed every other day. 

### 4.7. Transepithelial Electrical Resistance Measurements

The barrier function of AHSEs and VHSEs with 100% collagen or 50/50 C/KTN dermal compositions were measured by determining the transepithelial electrical resistance (TEER) of the differentiated keratinocyte stratum corneum layer using EndOhm tissue resistance measurement chambers (World Precision Instruments, Sarasota, FL, USA). Briefly, the transwell insert with each sample was placed inside the measurement chamber and immersed in Ca^2+^-free media. For the VHSEs, the hydrogel component was removed from the platform and placed in a 48 well transwell insert and, similarly to the AHSE, placed inside EndOhm tissue resistance measurement chambers designed for a 48 well insert. The electrode was then placed on top of the transwell, with a space of 1–2 mm between the top of the hydrogel and the bottom of the electrode. The electrical resistance was then measured. Each measurement was normalized to a blank transwell insert.

### 4.8. Permeability of Epidermal Barrier

The barrier function was also characterized by measuring the permeability of the stratum of the ASHE and the VHSE based on the fluorescence intensity of the 70 kDa FITC dextran that passed through the stratum corneum. This was done by measuring the fluorescence intensity of the media underneath the donor solution (underneath the transwell) of the AHSE and the VHSE at various time points (2, 3, 4, 8, 12, 24, and 48 h), following a protocol developed by Hsu et al. [62]. Briefly, for the AHSE, 200 µL of 0.1 mg/mL 70 kDa FITC dextran in differentiation media was placed on top of the sample inside the transwell insert and 2 mL of differentiation media without dextran was placed outside and underneath the transwell. For the VHSEs, the plastic coverslip on the bottom was removed to allow diffusion of the dextran solution (100 µL) on top of the epidermal barrier to the dextran-free solution underneath (3 mL). In both systems, the volume was adjusted to confirm that they were at the same level to avoid hydrostatic pressure. At each time point (1, 2, 3, 4, 8, 12, 24, and 48 h) the HSE systems were removed and the fluorescence intensity of the dextran in the bottom solution was read at Ex/Em 494/521 nm with the Cytation3 Multi-Mode plate reader. The samples were then placed back in the incubator for the next time point. To calculate the permeability coefficient, the fluorescence of the media was converted to the concentration of dextran using a standard curve. The concentration over time was plotted, and the slope of the linear segment of the plot was used to calculate the permeability coefficient using the following equation:

$$\;P_{d}=\frac{{dC}_{A}}{dt}\left(\frac{V_{A}}{C_{D}}\right)\frac{1}{A}$$

where *P_d_* is permeability coefficient, *dC_A_/dt* is the slope, *V_A_* is the volume of the donor medium (bottom of the sample), *C_D_* is the concentration of dextran in the top solution (0.1 mg/mL), and *A* is the area of the permeation surface. 

### 4.9. Endothelial Cell Growth

One hundred percent collagen and 50/50 C/KTN hydrogels were fabricated in the same way as discussed in previous sections. A total of 100 μL of hydrogel solution was seeded in individual 24 well plates (*n* = 3 or 4) and, after polymerizing for 1 h, 0.5 × 10^6^ TIME cells/cm^2^ were seeded on top. After day 1 post-seeding, TIME cells were stained with Hoechst 333422 (Thermo Fisher Scientific, Waltham, MA, USA) at a concentration of 1 μg/mL in the endothelial complete media and were incubated for 10 min, rinsed, and imaged. This Hoechst stain lasted for 3 days, and each sample was imaged up to 3 days post-seeding. Total confluency was measured by adjusting to a constant threshold for all samples using ImageJ software (Windows ij45-win-java8) and measuring the percentage of the image occupied by the cells.

### 4.10. Vessel Permeability Measurements 

Vessel permeability of the vascularized HSE was measured by injecting 70 kDa FITC dextran and monitoring mean fluorescence intensity of the dextran permeabilized through the vessel over the course of 30 min to show the functionality of the vessel and whether the lack of KTN (100% collagen) created a leakier vessel. Before injection of the dextran solution, an image of the vessel and the surrounding collagen was taken to measure the mean background fluorescence intensity to subtract from the dextran intensity after injection. A total of 15 µL of 0.1 mg/mL 70 kDa FITC dextran (Sigma-Aldrich, St. Louis, MO, USA) in 1× PBS was injected into the vessel and images were taken every 2 min for 30 min at an Ex/Em of 494/521 nm (FITC, dextran) and 588/635 nm (mKate, TIME cells of vessel) with the Zeiss Axio Observer inverted microscope (Zeiss, Oberkochen, Germany) at 5X magnification. Mean FITC fluorescence intensity within 2 regions-of-interest (ROIs) lining each side of the vessel in the outer hydrogel region was determined using ZEN^®^ 3.4 microscopy software at each time point. The following equation was used the calculate the permeability coefficient (*P_d_*):

$$P_{d}=\frac{1}{I_{1}-I_{b}}\left(\frac{I_{2}-I_{1}}{∆t}\right)\frac{d}{4}$$

where *I*_1_ is the first time point meant fluorescence intensity in the ROI, *I_b_* is the background intensity, Δ*t* is the time elapsed, *I*_2_ is the mean fluorescent intensity after Δ*t*, and *d* is the diameter of the vessel (711 µm). The *P_d_* of each time point was then averaged to determine the mean *P_d_*. Results were compared between vascular HSEs made of 100% collagen and 50/50 C/KTN. 

### 4.11. Histological Staining

To observe the structure, epidermal thickness, and level of differentiation, AHSEs and VHSEs were processed for histology and stained with hematoxylin & eosin (H&E) and immunohistochemistry (IHC staining). AHSEs and VHSEs were fixed with 4% PFA for 24–48 h after 2 weeks of differentiation at the ALI and were processed for histology and subsequent H&E and IHC staining for differentiation markers, including CK10, CK14, and involucrin. Samples were processed, embedded in paraffin, cut into 5 µm thick sections, and mounted onto glass slides. Slides were then deparaffinized, dehydrated, and either stained for H&E or stained using immunohistochemistry. For immunofluorescence staining, slides were blocked, permeabilized, and incubated with either anti-CK14 antibody (Abcam, ab181595, Cambridge, UK) at a 1:2000 dilution and anti-CK10 antibody (Abcam, ab9026, Cambridge, UK) at a 1:1000 dilution as a double stain in a BSA solution, or with anti-involucrin antibody (Abcam, ab181980, Cambridge, UK) at a 1:250 dilution in a BSA solution as a single stain for 1 h at room temperature. Slides were then washed with 1X HBSS and incubated with corresponding secondary antibodies (donkey anti-rabbit AF488 at 1:500 dilution in BSA for involucrin and CK14 and donkey anti-mouse AF594 at 1:500 dilution in BSA for CK10) for 1 h at room temperature and protected from light followed by additional 1× HBSS washes. Negative controls included human skin stained with the secondary antibodies. Slides were mounted with Prolong Gold Antifade Mountant with DAPI (Life Technologies, Carlsbad, CA, USA), cover-slipped, and imaged with the Axio Scan slide scanner (Zeiss, Oberkochen, Germany) at 20× magnification. 

### 4.12. Statistical Analysis

All data are presented as average ± SD. A student’s *t*-test or a two-way ANOVA was performed on GraphPad Prism software (Windows version 10.2.2) to determine the significance between different groups of hydrogel formulation and avascular vs. vascular with *p* < 0.05 were considered significant. A student’s *t*-test was performed for only dextran permeability through the stratum corneum and the endothelialized vessel. For TEER and dextran permeability measurements, *n* = 3 or 4; for TIME cell confluency, *n* = 3 or 4; for vascular permeability, *n* = 3; and for epidermal thickness, *n* = 15.

## 5. Conclusions

In this study, we were able to develop multiple skin models from the frequently used organotypic skin model (AHSE) to complex, multilayered vascularized skin models with a functional vessel (VHSE). Our AHSE model was able to demonstrate that the presence of KTN in the hydrogel increased barrier function by lowering the permeability of dextran through the developed epidermis and increasing the transepithelial electrical resistance. We were also able to show that the presence of vasculature in our 3D model increased the epidermal thickness compared to the AHSE static culture model, in which a dynamic flow was not employed. We were able to show the importance of a dynamic culture and vessel function in the formation of a multilayered skin model with proper keratinocyte differentiation. In future studies, this type of vascularized skin model can potentially be used for a more realistic immune response to introduce immune cells into the vessel or in wound healing studies, in which vessel permeability can be affected by wounding. In addition, with a fully stratified epidermal layer, this artificial skin may find utility in skin grafting as opposed to using artificial skin with incomplete formation of the epidermal stratified layer, as is present in static skin models. Transition of this vascularized model for drug testing and to the clinic will have potential for understanding and treating skin diseases. 

## Data Availability

The original contributions presented in the study are included in the article, further inquiries can be directed to the corresponding author.
